# Silica- Iron Oxide Nanocomposite Enhanced with Porogen Agent Used for Arsenic Removal

**DOI:** 10.3390/ma15155366

**Published:** 2022-08-04

**Authors:** Georgiana Mladin, Mihaela Ciopec, Adina Negrea, Narcis Duteanu, Petru Negrea, Paula Ianasi, Cătălin Ianași

**Affiliations:** 1 Faculty of Industrial Chemistry and Environmental Engineering, Polytechnic University of Timişoara, Victoriei Square, No. 2, 300006 Timisoara, Romania; 2 National Institute for Research and Development in Electrochemistry and Condensed Matter, 144th Dr. A. P. Podeanu Street, 300569 Timisoara, Romania; 3 Coriolan Drăgulescu’ Institute of Chemistry, Bv. Mihai Viteazul, No. 24, 300223 Timisoara, Romania

**Keywords:** arsenic, adsorption, sol–gel method, silica matrix, nanocomposite, iron oxide

## Abstract

This study aims to remove arsenic from an aqueous medium by adsorption on a nanocomposite material obtained by the sol–gel method starting from matrices of silica, iron oxide and NaF (SiO_2_/Fe(acac)_3_/NaF). Initially, the study focused on the synthesis and characterization of the material by physico–chemical methods such as: X-ray diffraction, FT-IR spectroscopy, Raman spectroscopy, atomic force microscopy, and magnetization. Textural properties were obtained using nitrogen adsorption/desorption measurements. The zero load point, pHpZc, was also determined by the method of bringing the studied system into equilibrium. In addition, this study also provides a comprehensive discussion of the mechanism of arsenic adsorption by conducting kinetic, thermodynamic and equilibrium studies. Studies have been performed to determine the effects of adsorbent dose, pH and initial concentration of arsenic solution, material/arsenic contact time and temperature on adsorption capacity and material efficiency. Three theoretical adsorption isotherms were used, namely Langmuir, Freundlich and Sips, to describe the experimental results. The Sips isotherm was found to best describe the experimental data obtained, the maximum adsorption capacity being ~575 µg As(III)/g. The adsorption process was best described by pseudo-second order kinetics. Studies have been performed at different pH values to establish not only the optimal pH at which the adsorption capacity is maximum, but also which is the predominantly adsorbed species. The effect of pH and desorption studies have shown that ion exchange and the physiosorption mechanism are implicated in the adsorption process. From a thermodynamic point of view, parameters such as ΔG°, ΔH° and ΔS° were evaluated to establish the mechanism of the adsorption process. Desorption studies have been performed to determine the efficiency of the material and it has been shown that the material can be used successfully to treat a real-world example of deep water with a high arsenic content.

## 1. Introduction

Water is vital to human existence [1,2]. Arsenic it is a metalloid that naturally occurs in soil, and is one of the most dangerous elements from the natural environment [3]. Usually, arsenite (${\mathrm{AsO}}_{3}^{3-}$) is much more dangerous than arsenate (${\mathrm{AsO}}_{4}^{3-}$). This element can be turned into different sulfur-containing ores, where it is merged with nickel, copper, cobalt, lead and other metals. Because of their relatively high solubility, arsenic and its derivatives are portable in the global environment [4]. Theresence of arsenic in water, especially in groundwater, was recognized decades ago as a significant problem. The safety risks and higher toxicology associated with arsenic presence in water were identified decades ago [5]. Arsenic in the natural environment has both anthropogenic and natural origins [6]. Due to anthropogenic activities, arsenic contamination has been widely recognized as one of the most significant impacts on the environment. Arsenic toxicity is the focus of many institutions, including industry, environmental agencies, and the general public. The elimination of arsenic has an indisputable significance for flora and fauna, for humanity, but also for ecosystem life overall.

The development of new drugs for serious diseases involves the usage of arsenic derivatives for most conventional medicines, for example, promyelocytic leukemia has been controlled by the use of arsenic trioxide. After ingestion, arsenic absorption occurs mainly at the intestinal tract level, while a limited penetration occurs as a result of rupture of the skin and inhalation [7]. Exposure to arsenic by inhalation, in addition to causing lung discomfort, vomiting and skin damage, is responsible for various conditions such as digestive discomfort, neuropathic pain, anemia, arterial damage and various forms of cancer—lung, skin, kidney, liver and bladder [8]. In case of poisoning, arsenic presence can damage and affect the internal organs of the body, and especially the skin, as well as affecting the body’s immunity [9]. Arsenic can enter the human body through inhalation, ingestion or dermal exposure. It is scattered throughout the body in a variety of glands, the liver, kidneys, lungs and skin [10,11].

In regions with intense industrial production, the air represents a major source of arsenic poisoning [12]. It is recognized that low levels of arsenic in drinking water produce substantial harmful effects, emphasizing the relevance of arsenic removal. New water quality legislation under the European Environment Protection Agency is stricter and includes a reduction in the concentration of arsenic in drinking water down from 50 ppb to 10 ppb [13].

The natural arsenic pollution of groundwater occurs in different areas: in indoor or closed basins, in sparsely populated regions or quasi-regions, and groundwater-containing alluvial deposits [14].

During the last decades, numerous techniques and processes able to remove arsenic from the environment have been developed and presented. Some of them are adsorption processes, electrocoagulation, nanoredimediation, ion exchange, electrokinetic processes, phytoremediation, membrane technology, chemical precipitation coagulation, precipitation and flocculation, bioremediation, ozone oxidation, electrochemical treatments and phytobial remediation [5,15,16,17,18,19,20,21,22].

Adsorption represents a valuable method able to reduce the amount of aqueous contamination with arsenic in the environment [1,23]. While activated charcoal is a cheap, long-lasting, environmentally safe adsorbent with a high ability to remove harmful elements from water, including arsenic [24,25,26], outstanding results have been recorded using nanoadsorbents [27,28,29], materials based on TiO_2_ or iron oxides [30]. Arsenic removal from polluted water often uses nanozerovalent iron that can be coupled with many other techniques such as Fenton and/or other methods, although more specific studies on the immobilization function as well as several technological advances still need to be carried [31]. Unlike classical adsorbent materials, new innovatively produced materials such as metal organic frameworks, nanotubes, graphene oxide and various other synthetic materials, represent a very good alternative for the efficient removal of both As(V) and As(III) ions, determined by very good partition coefficient and by an improved reuse and recycling [32,33]. Some attractive materials used as adsorbents for the recovery of arsenic-contaminated water are represented by hydrothalcites [34].

The aim of this study is to remove arsenic from the aqueous medium by adsorption on a nanocomposite material obtained by the sol–gel method starting from silica, iron oxide and NaF-(SiO_2_/Fe(acac)_3_/NaF). Nanoparticles are of great interest since they can be utilized in multiple application. Beside their properties, Fe-based nanoparticles are affordable and stable under extreme conditions and exhibit a polar surface, which is also the main reason for its applicability in catalysis, data storage devices and environmental remediation [35,36,37,38]. The applications regarding the arsenic absorption are successfully achieved due to their surface area and porosity, which can be manipulated by the synthesis parameters. To our knowledge, the present material has not been studied so far in this combination, highlighting the novelty of the research and as well as their potential in adsorption.

## 2. Materials and Methods

### 2.1. Synthesis and Characterization Material SiO_2_/Fe(acac)_3_/NaF

To obtain the nanocomposite (SiO_2_/Fe(acac)_3_/NaF), sol–gel method was used. Thus: (i) a first solution of Fe(acac)_3_ is obtained by dissolving 4 g of Fe(III) acetylacetonate (Sigma–Aldrich, St. Louis, MO, USA) in 50 mL methanol (Chimopar, SC CHIMOPAR TRADING SRL, București, Romania) by mechanical stirring at 50 °C for 30 min; (ii) the second solution is obtained by dissolving in 10 mL of distilled water the silica precursor, 10 mL of tetraethyl-orthosilicate-TEOS, Si(OC_2_H_5_)_4_, (Sigma–Aldrich). The second solution was introduced over solution 1. It was mixed for 2 h at 50 °C at 400 rpm with the addition of a mixture of 0.5 g of sodium fluoride, NaF (Sigma–Aldrich), the gelling material was released as a release agent. After a week of aging process, crystals formed in the form of needles. The material has been dried at 100 °C for 24 h, and further heat treated at 200 °C. To provide information about the structural fingerprint by which the molecules of the material can be identified, the Raman spectrum was made using the Shamrock 500i Spectrograph (Andor, Belfast, UK) at room temperature using laser excitation at 514 nm. At the same time, the material was characterized by Fourier transform infrared spectroscopy, FT-IR, of JASCO FT/IR-4200 apparatus (SpectraLab, Shimadzu, Kyoto, Japan).

The material was also analyzed by atomic force microscopy (AFM). AFM images were obtained by using Scanning probe microscopy platform (MultiView-2000 system, Nanonics Imaging Ltd., Jerusalem, Israel), at room temperature (298 K), in normal conditions, using system intermittent mode. The analysis used a chromium doped tip with a 20 nm radius and 30–40 kHz resonance. Further, in order to highlight material morphology and to prove the compound formation, new prepared adsorbent material was characterized by scanning electron microscopy (SEM) coupled with energy dispersive X-ray spectroscopy (EDX) using a FEI Quanta FEG 250 scanning electron microscope.

The zero-load charge, pZc, has been determined by bringing the studied adsorbent system into equilibrium. In this case, 0.1 g of SiO_2_/Fe(acac)_3_/NaF material was mixed with 25 mL of 0.1 N KCl solution at 200 rpm and a temperature of 298 K, using a Julabo SW23 thermostated and stirred water bath. The pH of the KCl solutions was adjusted in the range of 2–12, using NaOH solutions with a concentration of between 0.05 N and 2 N or HNO_3_ solutions with a concentration of between 0.05 N and 2 N. Further, each sample was filtered and subsequently resulting solution pH was determined using a pH meter of the METTLER TOLEDO, Seven Compact, S 210 type.

### 2.2. Arsenic Adsorption Studies

In the present paper, the influence of the solution pH on the adsorption process of As(III) on SiO_2_/Fe(acac)_3_/NaF synthesized material, varying the pH, in the range 2–14, was determined. Thus, 0.1 g of material was contacted for 60 min at 298 K with a 25 mL solution of As(III) (As_2_O_3_, Fluka, pa, ≥99.5%) of initial concentration, C_0_ = 100 µg As(III)/L. During these experiments, the pH has been adjusted using HNO_3_ and NaOH solutions with concentrations in the range of 0.1–1 N, obtained by diluting 63% HNO_3_ (Carl Roth) and NaOH, pellets (Sigma–Aldrich, pa, ≥99.5%).

In order to determine the influence of contact time and temperature on the adsorption capacity of SiO_2_/Fe(acac)_3_/NaF synthesized material, 0.1 g of material was accurately weighed, and to which 25 mL of As(III) solution of initial concentration 100 µg/L was added. The samples were stirred and thermostated for different time periods (15, 30, 45, 60 and 90 min) in a water bath, and at different temperatures (298 K, 308 K, 318 K and 328 K), at 200 rpm.

To determine the effect of the initial concentration of As(III) on the adsorption capacity of the material SiO_2_/Fe(acac)_3_/NaF, solutions of As(III) concentrations of 100, 200, 400, 800, 1000, 2000, 3000 and 5000 µg/L were prepared. Working concentrations were obtained from a stock solution of As_2_O_3_ (1000 mg/L) by appropriate dilution. Adsorption studies were performed at a temperature of 298 K for 60 min, by keeping the solution pH between 7 and 8. As(III) residual concentration was measured using the graphite furnace atomic absorption spectrophotometer, AA 6800, Schimadzu (Kyoto, Japan).

In all the experiments, adsorption capacity of the used sorbent, q (mg g^−1^), has been determined by using the following equation:

$$q=\frac{\left(C_{0}-C_{f}\right)V}{m}$$

where: C_0_—initial concentration of As(III) from solution, (mg L^−1^); C_f_—residual concentration of As(III) from solution, (mg L^−1^); V—volume solution, (L); m—adsorbent mass, (g).

### 2.3. Arsenic Desorption Studies

To determine the efficiency of the SiO_2_/Fe(acac)_3_/NaF material, regeneration studies were performed. Thus, 1 g of depleted material, following the process of adsorption of As(III), was stirred with 25 mL of 5% HCl. Residual As(III) concentration was measured using the graphite furnace atomic absorption spectrophotometer, AA 6800, Schimadzu.

### 2.4. Arsenic Removal from Real Ground Water

Starting from the adsorbent performance of the studied material, studies were performed on the removal of arsenic from real water in a static regime, using 2 g of SiO_2_/Fe(acac)_3_/NaF over which a sample of real water was passed. Real water used during present study have the composition presented in Table 1. The following parameters are established according to the standardized methods, specific to the determination of the drinking water quality. Ca^2+^, Mg^2+^, Na^+^, K^+^, Fe^n+^ and Mn^n+^ were determined by atomic absorption spectrometry using the Varian Spectra AAS 280 FS spectrometer. ${\mathrm{NH}}_{4}^{+}$, ${\mathrm{NO}}_{2}^{-}$, ${\mathrm{NO}}_{3}^{-}$, ${\mathrm{SO}}_{4}^{2-}$ ions were determined by UV-Vis spectroscopy using the Varian Carry 50 spectrophotometer. The As(III) concentration was determined using the graphite furnace atomic absorption spectrophotometer, AA 6800, Schimadzu.

## 3. Results and Discussion

### 3.1. Synthesis and Characterization Material SiO_2_/Fe(acac)_3_/NaF

#### X-ray Diffractogram

**Figure 1:**
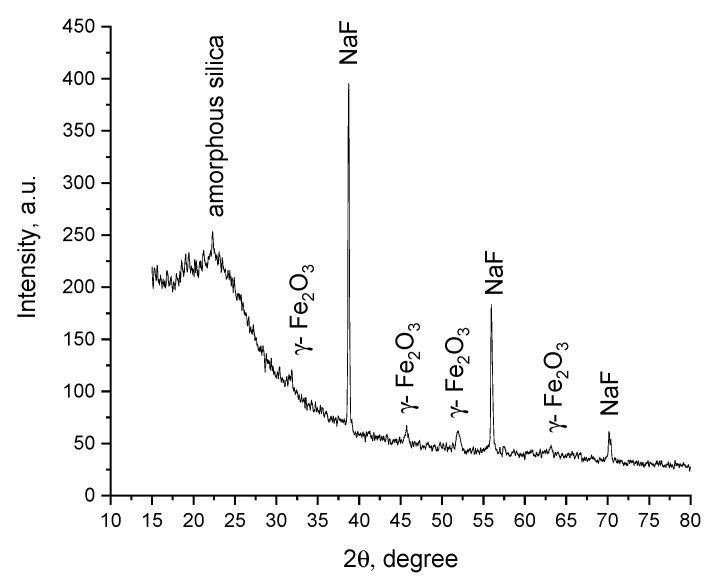
XRD of SiO_2_/Fe(acac)_3_/NaF.

The X-ray diffractogram is shown in Figure 1.

Analyzing the data presented in Figure 1 demonstrates the presence of three different phases. Peaks observed at 38, 53 and 70 degrees are specific for NaF phase (reference code 00-001-1184). Peaks located at 32, 38, 45, 52, 63 are specific to the maghemite phase [39]. From comparisons with the literature [39], it can be observed that the specific peaks for the maghemite are slightly shifted to smaller angles. In order calculate the crystallite size was used the Scherrer equation, and for new synthesized SiO_2_/Fe(acac)_3_/NaF compound was founded that the mean crystallite size is 36 nm. The result indicates that the doping of materials with different ions metals influence the structure.

### 3.2. Raman and Infrared Spectroscopy

**Figure 2:**
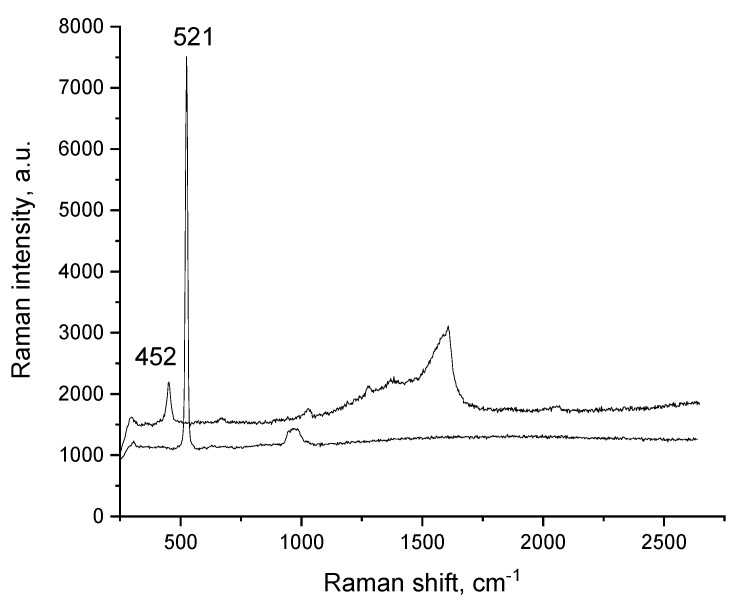
Raman spectra for SiO_2_/Fe(acac)_3_/NaF.

Taking into account that the sol–gel process implies hydrolysis and condensation, Raman spectroscopy is a useful tool for determining the changes in the material [40]. Taking into account the data presented in the literature, the most prominent peak is located around 1600 cm^−1^ as observed in our obtained spectra (Figure 2) [41].

The aforementioned band is attributed to the metal–acetylacetonato compounds with their specific C=O and C=C stretching vibration bonds, which have lower or higher value in dependence of the predominant bond placed between 1605–1560 cm^−1^ or around 1640 cm^−1^. Due to its specific and complex structure, the other acetylacetonato-specific peak is located at 1380 cm^−1^ indicating the C-H stretching vibration bond. Due to the C=C bonds, 1540 cm^−1^ and 1290 cm^−1^ bands are expected. In our spectra the 1540 cm^−1^ band is present as a broad low intensity shoulder whereas the 1290 cm^−1^ band is shifting from 1275 to 1283 cm^−1^. Krishnan et al. attributed the 1600 cm^−1^ band to the C–O stretching mode of the free enol form of acac [42,43]. The metal–oxygen stretching bonds are expected in the 450 cm^−1^ region, as indicated in the literature [42,43]. The 566–582 cm^−1^ may also be attributed to the metal–oxygen bonds [44]. The different intensity in the 566–582 cm^−1^ area associated with the M–O bond takes place as a result of different gelation and precipitation degree. Peaks located at 664–672 and 566–582 cm^−1^ are associated with the presence of Si-O bonds as explained in the literature [45], which can explain the inclusion of the silica in the structure of the new prepared adsorbent material. The decrease in the crystallite size is indicated by the broadening, asymmetry and red-shift of the peaks, a phenomenon that is visible when comparing the silica and the material’s final spectrum [46].

### 3.3. FT-IR Spectra

**Figure 3:**
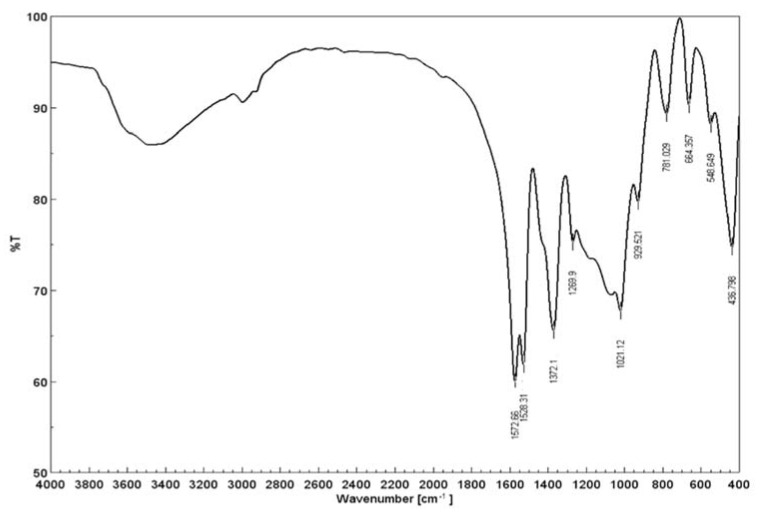
FT-IR spectra for SiO_2_/Fe(acac)_3_/NaF.

Regarding the FT-IR analysis (Figure 3) the following bands were observed: 1572, 1528, 1372, 1269, 1021, 929, 781, 664, 548, 436 cm^−1^.

Taking into account the similarities between the Raman and FT-IR, some of the vibrations are expected to tale place at the same spectral location, whereas some may also be absent. It is clear that the 430–460 cm^−1^ vibrations are assigned to the metal–oxygen bonds, almost at the same location as observed in the Raman spectra. The 548 cm^−1^ peak is also shifted in comparison to the Raman spectra, indicating the Si–O bonds. IR features found at 1572 cm^−1^ (O–H), 1372 cm^−1^ (CH_3_), the shoulders at 1207 cm^−1^ and 1529 cm^−1^ (C=C), 1021 cm^−1^ (C–C) and 664 cm^−1^ (O–H) were attributed to the acetylacetonate organic part [47]. The 795–780 cm^−1^ are assigned with the M–O bonds since it is well known that coordination complexes are difficult to detect due to the influence of metal coupling, which changes the vibration modes of the compound as the final result [42].

### 3.4. Morphological Study with Atomic Force Microscopy

**Figure 4:**
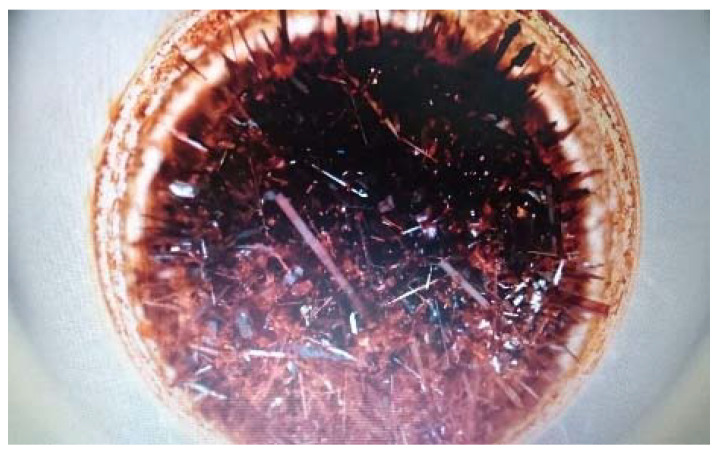
Image of crystals for SiO_2_/Fe(acac)_3_/NaF.

**Figure 5:**
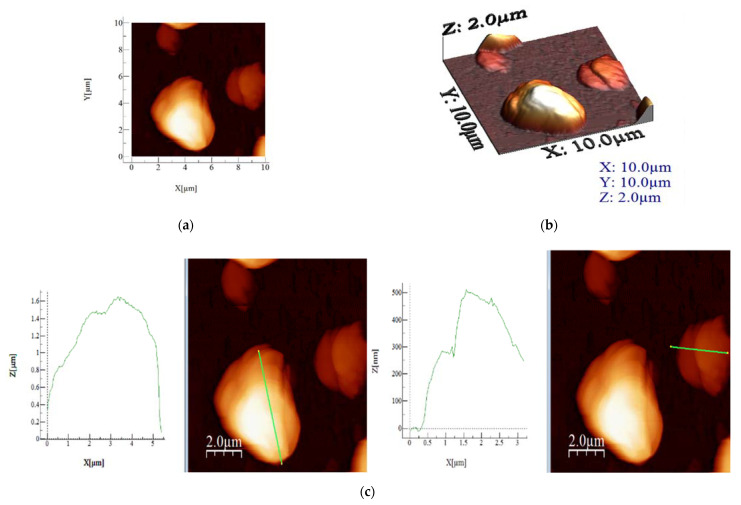
AFM images (**a**), 3D images (**b**) and height profile (**c**) of material on scale 10 µm × 10 µm.

Figure 4 presents the obtained crystals before the grinding, followed by the AFM images, and the 3D and height profile of material on the scale 10 µm × 10 µm (Figure 5) which were performed only for the grinded material. AFM analyses are useful for the surface determination, whereas the more homogenous materials with high porosity are better for the electrical measurements.

Scanning on a 5 µm × 5 µm scale was performed for closer observation of the round-shaped formations of material. Some differences were expected regarding the rugosity of materials in comparison to the 10 µm × 10 µm scale images, due to the more specific local area of analysis. Nonetheless, the 10 µm × 10 µm images should be considered more reliable, as they contain more features on a greater scale. This class of analysis material still has the one with the highest roughness values, as well as highest Sp, Sy and average height. It confirms the fact that this compound has the largest round formations, which is also visible in the height profile of the selected area.

**Figure 6:**
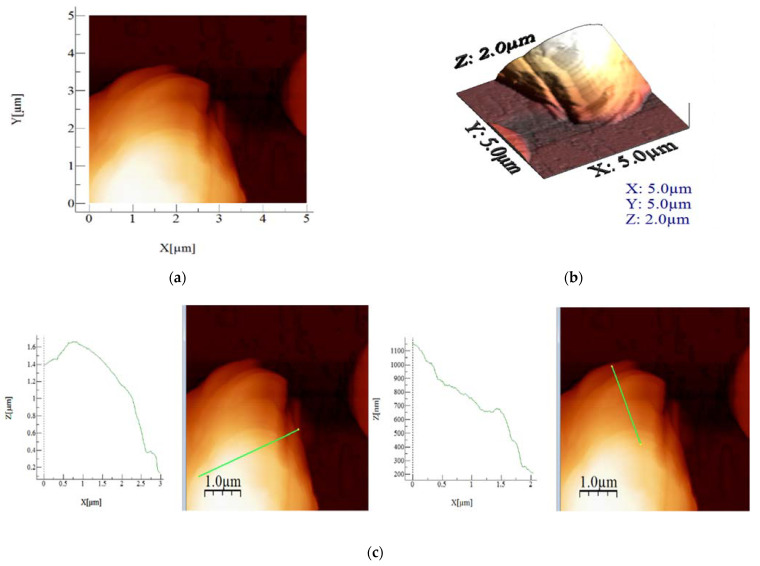
AFM images (**a**), 3D images (**b**) and height profile (**c**) of material on scale 5 µm × 5 µm.

Based on the recorded AFM images, the values for average roughness (Sa), mean square root roughness (Sq), maximum peak height (Sp), maximum valley depth (Sv), maximum peak-to-valley height (Sy) and average height were calculated, which are presented in Table 2. The images were scanned on a scale of 10 µm × 10 µm (Figure 5a) and 5 µm × 5 µm (Figure 6a), providing insight into the particularities of each sample. From the obtained results, highest roughness values (both Sa and Sq) were obtained. Interestingly, that material also has the maximum peak to valley height (Sy) and also highest maximum peak height (Sp) values.

### 3.5. Nitrogen Adsorption–Desorption Isotherms

**Figure 7:**
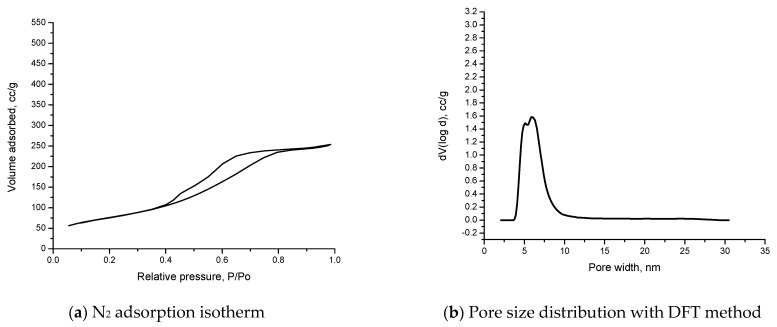
N_2_ adsorption isotherm (**a**) and pore size distribution (**b**) of material SiO_2_/Fe(acac)_3_/NaF.

In order to observe the differences in textural structure, the nitrogen adsorption–desorption isotherms are performed. Figure 7 presents the N_2_ isotherm of the material.

After analyzing the data, the materials obtained indicate a type IV isotherm. The isotherm indicates that capillary condensation occurs and that the pore exceeds a critical width.

In Figure 7b, the pore size distribution is presented. The data parameter obtained indicates only mesoporous material in our case.

In Table 3, the textural parameters are presented.

### 3.6. Magnetic Measurements

**Figure 8:**
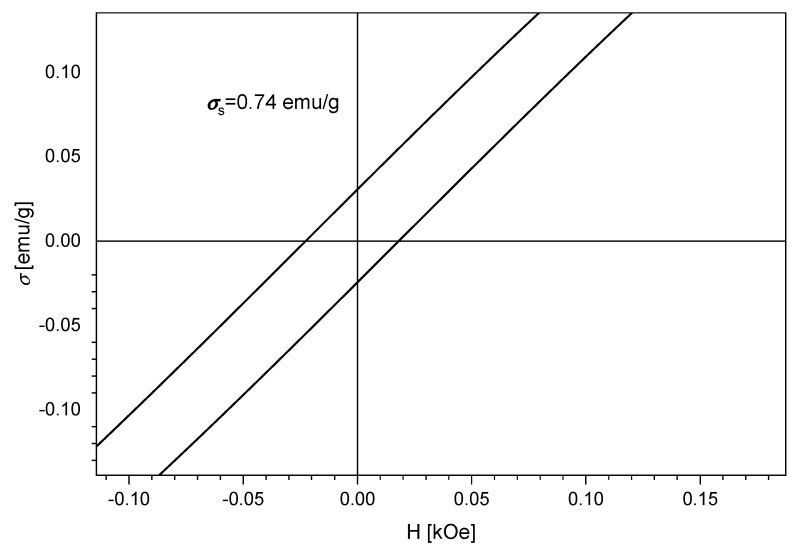
Measured magnetic saturation.

Figure 8 indicates the magnetic saturation curve with the extracted data using a homemade induction magnetometer with an AC field of 50 Hz and amplitude of 5 kOe. The fitting of the hysteresis loop branch was performed with Langevin-type transition functions [48].

By analyzing the results displayed in Figure 8, we observed that our material presents superparamagnetic behavior. The total saturation magnetization indicates a value of 0.74 emu/g. The remanent magnetization obtained indicates a value of 0.03 [emu/g] with a coercive field (H) of 0.01 [kOe].

### 3.7. Point of Zero Charge Determination (pH_pZc_)

**Figure 9:**
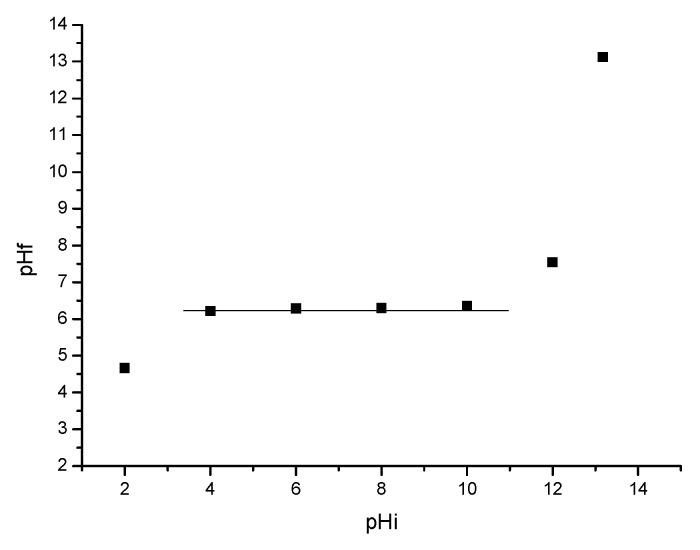
Determination of pH_pZc._ for new synthesized material.

Figure 9 shows the relationship between the final pH and initial pH, establishing the pH_pZc_ value of the material.

The point of zero charge (pzc) represents the value of the pH where the net charge of the material surface (i.e., adsorbent’s surface) is equal to zero at some specific ambient temperature, aqueous solution composition, and applied pressure [49].

In the case of SiO_2_/Fe(acac)_3_/NaF, the pH_pZc_ is 6.3. If we work at pH~6, the As(III) species that are adsorbed can be neutral. If the pH is worked out in the range of 6–8 species, they can also be anionic, as the surface of the material is positively charged.

### 3.8. Scanning Electron Microscopy Characterization

**Figure 10:**
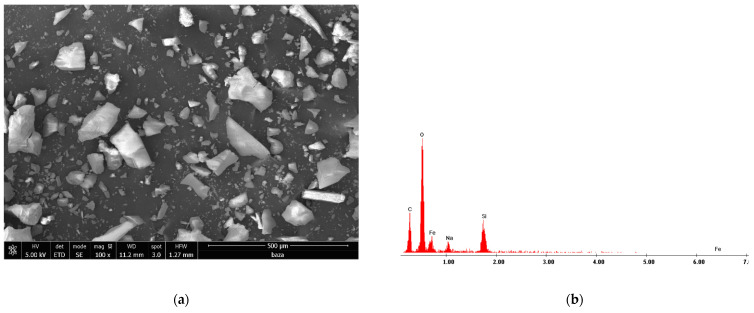
(**a**) SEM micrograph; (**b**) EDX spectrum.

To highlight the morphology of the new prepared adsorbent material, the SEM micrograph as recorded (Figure 10a). Furthermore, in order to prove the formation of the desired adsorbent material, the EDX spectrum was recorded (Figure 10b).

Analyzing data presented in Figure 10a, we can observe that the obtained adsorbent material presents a relatively homogenous structure, with well-defined crystals. Moreover, the EDX spectra confirm the formation of the desired adsorbent material.

### 3.9. Arsenic Adsorption Studies

#### 3.9.1. pH Effect

**Figure 11:**
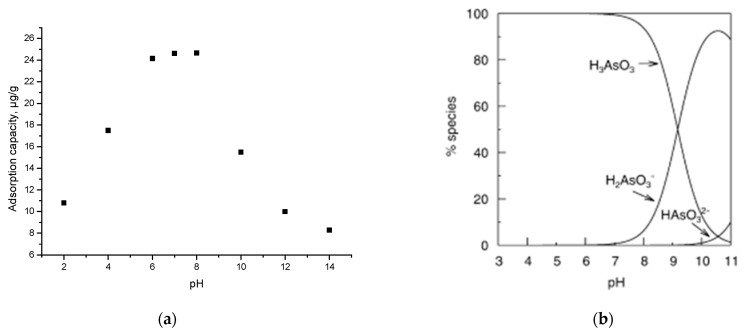
pH (**a**) and speciation as a function of pH (reprinted with permission from [14]) (**b**).

The data presented in Figure 11a shows how the maximum adsorption capacity of As(III) is influenced by the evolution of pH.

Thus, at pH < 6, the adsorption capacity increases with increasing pH. In the pH range 6–8, the adsorption capacity reaches maximum values (~24 µg As(III)/g material). At pH > 8, the adsorption capacity decreases.

Figure 11b presents the distributions of the As species as a function of pH. The species specific to the optimal pH of the adsorption process of As(III) on the SiO_2_/Fe(acac)_3_/NaF material are H_3_AsO_3_ and/or H_2_AsO_3_^−^ [14].

The fact that the optimal pH coincides with the pHpZc of the material demonstrates that the process of removing As(III) by adsorption will proceed with good efficiency.

**Figure 12:**
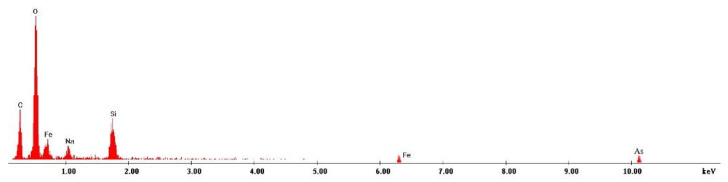
EDX spectrum recorded for the exhausted adsorbent material.

In order to prove that the new prepared material is able to adsorb the As(III) ions from the used aqueous solutions, we recorded the EDX spectrum for the exhausted adsorbent material (spectrum depicted in Figure 12). In this spectrum, the presence of the As(III) specific peak can be observed, confirming in this way our supposition that the new prepared material is suitable for As(III) removal.

#### 3.9.2. Influence of the Adsorbent Dose

**Figure 13:**
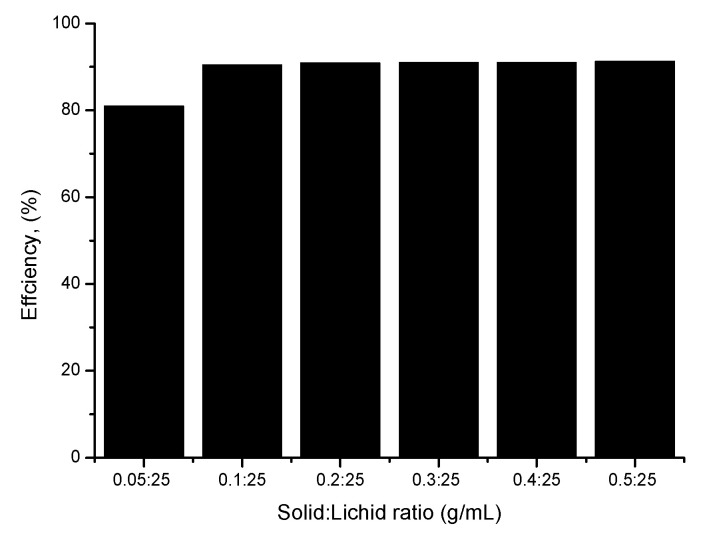
Effect of solid:liquid ratio.

In order to establish the optimum conditions for As(III) ions removal, the influence of the adsorbent dose has been studied. In this context, different amounts of adsorbent material were weighed and mixed with 25 mL of arsenic solution. Obtained dates are presented in Figure 13.

Analyzing the information presented in Figure 12, it can be observed that by increasing the solid:liquid ratio from 0.05:25 at 0.1:25 g/mL, the adsorption efficiency can be increased from 80% at 91%. Any further increase in the solid:liquid ratio does not lead to any significant increase in the adsorption efficiency. Based on this observation, further experiments were carried out using 0.1:25 g/mL solid:liquid ratio.

#### 3.9.3. Contact Time Effect

The adsorption process is determined by the contact time and temperature, knowledge of these two parameters being very important in addition to the pH of the solution with As(III) ions and the concentration of As(III) ions in the solution.

**Figure 14:**
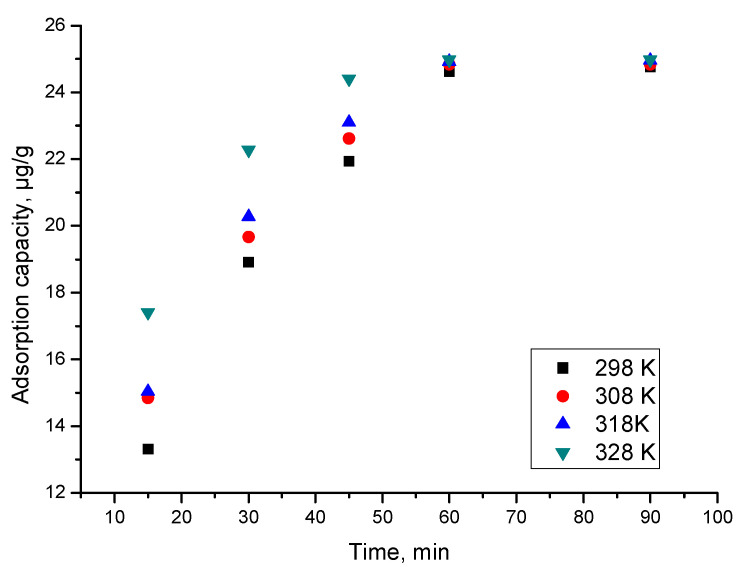
Contact time effect at different temperatures.

The role of contact time and temperature is shown in Figure 14.

From the data presented in Figure 14, it can be observed that as the contact time increased, so did the adsorption capacity. After 60 min, the adsorption capacity remains approximately constant, meaning that a further increase in contact time is not justified.

Based on the fact that the adsorption capacity increased with the temperature increase, it can be deduced that the adsorption process of As(III) is influenced by temperature. However, it can also be seen that the increase in adsorption capacity with temperature increase is not significant, and further studies at temperatures above 298 K are not warranted.

### 3.10. Kinetic Studies

In order to investigate the kinetics of the adsorption process of As(III) on SiO_2_ Fe(acac)_3_/NaF material, the obtained experimental data were modeled using the pseudo-first-order and pseudo-second-order kinetic equation.

Kinetic equations used to describe the pseudo-first-order model (Lagergren model) is [50]:

$$\ln\;\left(q_{e}-q_{t}\right)={\mathrm{lnq}}_{e}-k_{1}t$$

where: q_e_—equilibrium adsorption capacity, µg/g; q_t_—adsorption capacity at t time, µg/g; k_1_—speed constant for pseudo-first order equation, 1/min; t—contact time, min.

For the pseudo-second-order one (model Ho and McKay) is [51]:

$$\frac{t}{q_{t}}=\frac{1}{k_{2}q_{e}^{2}}+\frac{t}{q_{e}}$$

where: q_e_—equilibrium adsorption capacity, µg/g; q_t_—adsorption capacity at t time, µg/g; k_2_—speed constant for pseudo-second order equation, g/µg∙min; t—contact time, min.

For the pseudo-first-order equation, the linear dependence ln(q_e_ − q_t_) = f(t) was plotted, and from the equation k_1_ and q_e,calc_ were calculated.

For the pseudo-second-order equation the linear dependence t/q_t_ = f(t) was plotted. From the equation of the line, k_2_ and q_e,calc_ were evaluated.

**Figure 15:**
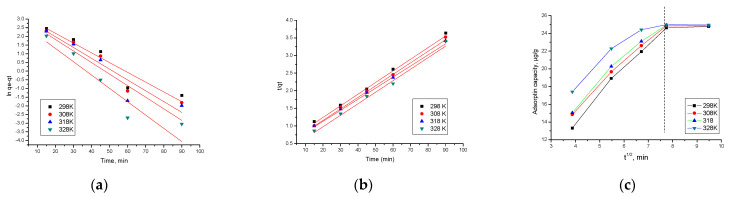
Kinetic studies of As(III) adsorption. (**a**) Pseudo-first-order; (**b**) Pseudo-second-order; (**c**) intraparticle diffusion.

The kinetics of the adsorption process of As(III) on SiO_2_/Fe(acac)_3_/NaF material at different temperatures were studied. In Figure 15a,b pseudo-first-order and pseudo-second-order isotherms obtained at four different temperatures are shown.

In addition to the typical kinetic models, pseudo-first-order and pseudo-second-order, the intraparticle diffusion is also studied in this paper.

In order to distinguish whether film diffusion or intraparticle diffusion represent the speed determinant stage, the experimental data were modeled according to the Weber and Morris model [52]:

q_t_ = k_diff_·t^1/2^ + C

where: q_t_—adsorption capacity at t time, µg/g; k_diff_—speed constant for intraparticle diffusion, µg/g·min^1/2^; C—constant correlated with the thickness of the liquid film surrounding the adsorbent particles.

Figure 15c shows the models for intraparticle diffusion at four different temperatures.

The values of speed constants, of the calculated adsorption capacity, as well as the values obtained for the k_diff_ and C parameters, following the modeling are presented in Table 4. The values of the regression coefficient, R^2^ are also presented in the same table.

Data presented in Table 4 demonstrate that the experimental data are modeled very well according to the pseudo-second-order kinetic model. This is supported by the value of the regression coefficient, R^2^~1 (0.9930–0.9973). In the case of modeling data according to the pseudo-first-order kinetic model, R^2^ is between 0.9076 and 0.8333. Furthermore, q_e,calc_ based on the pseudo-second-order isotherm has values close to q_e,exp._ Temperature influences the values of the parameters k_2_, q_e,calc_, but not significantly, meaning that we consider it necessary to work at temperatures higher than 298 K.

At the same time, it can be observed from Figure 15c that the intraparticle diffusion graph present a multi-linearity of the As(III) adsorption, signifying that the entire adsorption process, can be divided into two different stages [53,54]. Based on the model proposed by Webber and Morris, if the intraparticle diffusion represents the limiting step, the dependence of q_t_ = f(t^1/2^) must be a straight line passing through the origin [54,55]. The more pointed part of the graph is associated with the As(III) ions diffusion from the solution to the external surface of the used adsorbent. The second part of the adsorption process is associated with the adsorption process where the rate-limiting factor is the intraparticle diffusion process [54,55].

The adsorption rate is correlated with the slope of each linear segment presented in Figure 15c, a higher slope being correlated with a fasted adsorption of As(III) onto the studied adsorbent material [53,56]. From this figure, it can be observed that the slope of the first linear dependence is higher than the slope of the second linear dependence, meaning that stage 1 of the adsorption represents the velocity determinant stage, and the intraparticulate diffusion represents the limiting speed rate in stage two due to the low availability of the As(III) ions into the solution [54,57]. From the data presented in Table 4, it can be observed that with increasing temperature, the k_diff_ value also increases.

### 3.11. Theromdinamic Studies

Thermodynamic studies were performed in the temperature range 298–328 K. The value of the Gibbs free energy was calculated using the Gibbs-Helmholtz equation [58].

$${\Delta G}^{\circ }={\Delta H}^{\circ }-T\cdot {\Delta S}^{\circ }$$

where: ΔG°—standard Gibbs free energy variation, kJ/mol; ΔH°—standard enthalpy variation, kJ/mol; ΔS°—standard entropy variation, J/mol∙K; T—absolute temperature, K.

Firstly, the values of standard variation of the entropy ΔS° and the standard variation of the enthalpy ΔH° were evaluated, from the linear dependence ln K_d_ = f(1/T) (based on van’t Hoff equation):

$$\ln\;K_{d}=\frac{{\Delta S}^{\circ }}{R}-\frac{{\Delta H}^{\circ }}{\mathrm{RT}}$$

where: K_d_—equilibrium constant; ΔS°—standard entropy variation, J/mol∙K; ΔH°—standard enthalpy variation, kJ/mol; R—the ideal gas constant, 8.314 J/mol∙K.

The equilibrium constant is the ratio between the adsorption capacity at equilibrium q_e_ and the equilibrium concentration C_e_.

$$K_{d}=\frac{q_{e}}{C_{e}}$$

**Figure 16:**
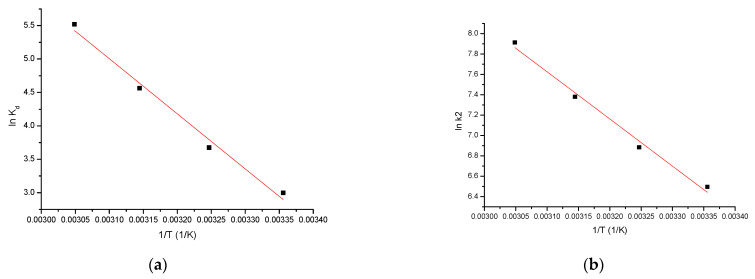
Thermodynamic studies. (**a**) ln K_d_ vs. 1/T; (**b**) ln k_2_ vs. 1/T.

In Figure 16a, the line ln K_d_ = f(1/T) will be represented.

For the adsorption of As(III) by the SiO_2_/Fe(acac)_3_/NaF material, the activation energy E_a_ was calculated, using the Arrhenius equation and the velocity constant from the pseudo-second-order kinetic model.

$$\ln\;k_{2}=\mathrm{lnA}-\frac{E_{a}}{\mathrm{RT}}$$

where: k_2_—Speed constant, g/min∙mg; A—Arrhenius constant, g∙min/mg; E_a_—Activation energy, kJ/mol; T—absolute temperature, K; R—The ideal gas constant, 8.314 J/mol∙K.

Th value of activation energy can provide information about the nature of the adsorption process, including whether it is physical or chemical. The value of the activation energy for the As(III) adsorption on SiO_2_/Fe(acac)_3_/NaF was calculated from the equation of the linear dependence between ln k_2_ and (1/T) (Figure 16b).

Table 5 shows the thermodynamic parameters resulting from the three temperatures.

From the resulting data, it can be observed that ΔH^0^ has a positive value, which means that the adsorption process is endothermic. At the same time, when the is value between 80 and 400 kJ/mol, the process can be considered physical [59].

It is also observed that ΔG^0^ has negative values and increases in absolute value with temperature increase, which indicates that the adsorption process is spontaneous and influenced by temperature. A positive value of ΔS^0^ indicates the favorability of the adsorption process, operating at the interface of the material SiO_2_/Fe(acac)_3_/NaF/solution and As(III).

It is observed that the activation energy (4.13 kJ/mol) is < 40 kJ/mol, which means that the adsorption process is physical in nature [59].

### 3.12. Initial Concentration Effect. Equilibrium Studies

**Figure 17:**
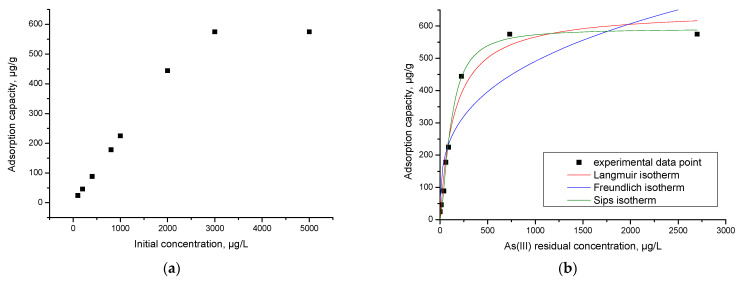
(**a**) Influence of the initial concentration influence. (**b**) Equilibrium studies.

The maximum adsorption capacity at equilibrium can be evaluated by taking into account the distribution of As(III) ions between the prepared adsorbent and arsenic aqueous solution. In this context, another parameter which influences the adsorption process is the initial concentration of the As(III) ions into the solution. In this case, the obtained experimental data are depicted in Figure 17a. In order to establish the adsorption mechanism, the obtained experimental data were modeled using three different isotherms: Langmuir, Freundlich and Sips. The Langmuir isotherm [60] assumes that the adsorbents have an ideal surface, and the adsorbate presents ideal gas behavior. Based on this model, the maximum adsorption capacity of the material by using the nonlinear expression can be determined by:

$$q_{e}=\frac{q_{L}K_{L}C_{e}}{1+K_{L}C_{e}}$$

where: q_L_—Langmuir maximum adsorption capacity, mg/g; K_L_—Langmuir constant.

The linearized form of the Langmuir isotherm used for modeling is:

$$\frac{C_{e}}{q_{e}}=\frac{1}{q_{L}K_{L}}+\frac{C_{e}}{q_{L}}$$

The Freundlich isotherm model assumes that the surface area of the material with adsorbent properties is heterogeneous, meaning that the heat distribution required for the adsorption process on the surface of the adsorbent material is uneven, and multilayer adsorption can occur due to unlimited active centers. The Freundlich isotherm is an empirical isotherm [61] given by the relationship:

$$q_{e}=K_{F}C_{e}^{1/n_{F}}$$

where: K_F_ and n_F_—characteristic constants that may be related to the relative adsorption capacity of the adsorbent and the adsorption intensity.

The linear shape of the Freundlich isotherm is:

$${\mathrm{logq}}_{e}={\;\mathrm{logK}}_{F}+1/n_{F}{\mathrm{logC}}_{e}$$

Sips Isotherm [62] was derived from the isotherms Langmuir and Freundlich. In the case of low adsorbate concentrations, Sips isotherm is reduced to that of Freundlich, and in the case of higher adsorbate concentration it is reduced to the Langmuir isotherm. Therefore, this isotherm can be used to calculate the adsorption capacity. The nonlinear equation of the Sips isotherm is:

$$q_{e}=\frac{q_{S}K_{S}C_{e}^{1/n_{S}}}{1+K_{s}C_{e}^{1/n_{S}}}$$

where: K_S_—constant related to the adsorption capacity of the adsorbent; n_S_—heterogeneity factor.

The isotherms obtained by the graphical representation of q_e_ = f(C_e_) are shown in Figure 17b.

Analyzing the obtained experimental data presented in Figure 17a reveals that the maximum adsorption capacity increases with the increase in the As(III) initial concentration until a maximum adsorption capacity (575.1 μg/g) was obtained.

The specific parameters of each isotherm used to model the experimental data are obtained from the slopes of the straight line and by using the ordinate from the origin (Table 6).

The dependence between the equilibrium concentration (C_e_) of As(III) and the adsorption capacity demonstrates that at the increase in the equilibrium concentration, the maximum adsorption capacity (q_e, exp_) also increases until equilibrium is reached.

According to the data in Table 6, the model that best describes the adsorption process is the Sips isotherm, because the regression coefficient, R^2^, is closest to 1 (R^2^ = 0.9916).

It is well known that the Sips model is a combination of the Langmuir and Freundlich models, which can be identified if: (i) the studied adsorption is an homogeneous process; (ii) adsorption occurs by the interaction of a solute molecule with an active centre from the sorbent surface; (iii) the sorbent surface contains a limited number of active centres; (iv) at equilibrium they are only partially occupied, regardless of the temperature at which the adsorption process is taking place; (v) the adsorption takes place on the surface of the sorbent resulting in a monolayer, the solute molecules being retained only on the sorbent-free surface; (vi) not all active centres on the surface of the sorbent are equal in energy; (vii) there may be some interactions between the solute molecules, and, therefore, once the sorbent surface is coated, additional solute molecules may still be adsorbed; and (viii) it can be used to describe the adsorption processes of solute molecules in multilayer [63]. The maximum adsorption capacity of As(III) on the adsorbent material was q = 575.1 µg/g for an initial concentration of C = 500 µg/L.

Based on data from the literature, a comparison of the material studied for the recovery of As(III) with other materials (Table 7) was showed that the SiO_2_/Fe(acac)_3_/NaF material has good adsorption capacity.

### 3.13. Arsenic Adsorption/Desorption Studies

**Figure 18:**
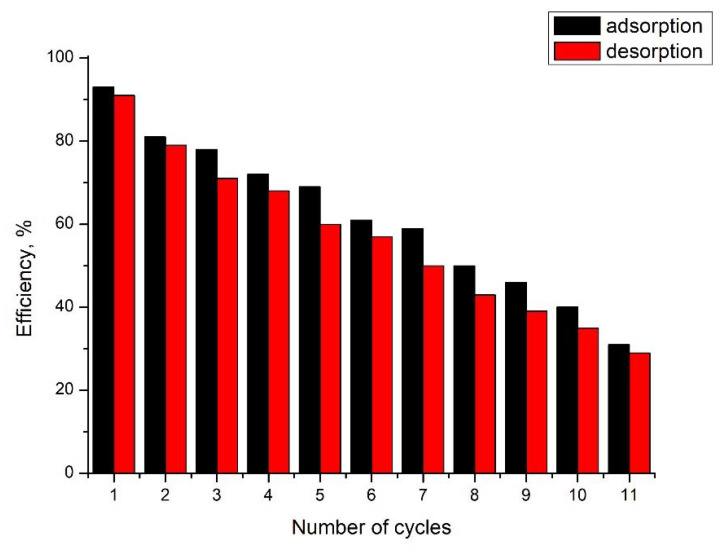
Adsorption/desorption cycles.

Desorption studies have shown that 93% of the adsorbed As(III) can be desorbed from the material using 5% HCl. Data regarding adsorption/desorption cycles are presented in Figure 18. Analyzing the data presented in Figure 18 allows us to observe that the newly produced adsorbent material can be reused with good efficiency for 11 adsorption/desorption cycles.

#### Arsenic Removal from Real Ground Water, Competing Ions Influence

From the obtained experimental data, it was found that the use of SiO_2_/Fe(acac)_3_/NaF material allows the removal of As(III) from real water (Table 8). From this, we can conclude that following the adsorption process, the content of foreign ions present in the water positively influences the process. Thus, the content of As(III) decreases reaching the limits allowed by the World Health Organization, WHO [70].

At the same time, the concentration of Mn^n+^ and Fe^n+^ decreases, reaching the limits allowed by the legislation.

## 4. Conclusions

In our study, we obtained a novel material based on SiO_2_, Fe(acac)_3_ and NaF as a porogenic agent, which was studied from the phase composition point of view, indicating that no impurities were found in the materials, as well as providing the proof that the bonds were formed between the compounds as observed in the Raman and FT-IR spectra. The AFM studies provided us the information regarding the surface of the material, indicating that our material is porous and formed layer-by-layer by sheets forming in round clusters. The surface area of SiO_2_/Fe(acac)_3_/NaF determined with BET method indicates a value of 276 m^2^/g. The magnetization saturation indicates a value of 0.74 emu/g.

The arsenic recovery process takes place under the following conditions: pH in the range of 6–8, contact time 60 min and temperature 298 K. Based on the results obtained, kinetic, thermodynamic and equilibrium studies were performed. By modeling the experimental data, we can observe that the pseudo-second-order isotherm model is the one that best describes the process.

To distinguish whether film diffusion or intraparticle diffusion is the determinant speed step, the kinetic experimental data were processed using the Weber–Morris model.

For SiO_2_/Fe(acac)_3_/NaF material, the porous structure allows the adsorption sites to be placed inside the surface of the adsorbent channels, which indicates that As(III) is adsorbed on the surface of SiO_2_/Fe(acac)_3_/NaF (step 1), and then the adsorption process reaches equilibrium (step 2), which means that the intraparticle diffusion is not a limiting step during adsorption. Adsorption is performed in the film. The activation energy, E_a_, was also determined by the fact that the adsorption process is of a physical nature.

Thermodynamic studies have established that the adsorption process is endothermic, spontaneous and influenced by temperature, and that the adsorption process takes place at the interface of the material SiO_2_/Fe(acac)_3_/NaF/solution with As(III). Following the equilibrium studies, the Sips model is the one that best describes the adsorption process, establishing the maximum adsorption capacity of SiO_2_Fe_x_O_y_ SiO_2_/Fe(acac)_3_/NaF material as 575.1 µg As(III)/g material. Desorption studies have confirmed that the adsorption process is controlled by pH, so that if the process proceeds at pH = 6–8, the material can be recovered and reused with high efficiency.

We can conclude that the SiO_2_/Fe(acac)_3_/NaF material can be successfully used for the recovery of As(III) from aqueous solutions, by adsorption. At the same time, the material can be used to obtain drinking water, which complies with the WHO recommendations in terms of arsenic content. Repeated cycles of adsorption/desorption may also be used.

## Figures and Tables

**Table 1:** Real water composition.

Parameters	Content
Ca^2+^ (mg/L)	89 ± 8.9
Mg^2+^ (mg/L)	44 ± 4.4
Na^+^ (mg/L)	120 ± 12
K^+^ (mg/L)	1.75 ± 0.17
Fe^n+^ (mg/L)	0.6 ± 0.06
Mn^n+^ (mg/L)	0.4 ± 0.04
P_total_, (mg/L)	5.6 ± 0.56
${\mathrm{NH}}_{4}^{+}$, (mg/L)	4.4 ± 0.44
${\mathrm{NO}}_{3}^{-}$, (mg/L)	12.3 ± 1.23
${\mathrm{NO}}_{2}^{-}$, (mg/L)	0.74 ± 0.07
${\mathrm{SO}}_{4}^{2-}$, (mg/L)	8.2 ± 0.82
As(III) (μg/L)	104 ± 10.4

**Table 2:** Values obtained from AFM analysis on scale of 5 µm × 5 µm.

Sample Name	Ironed Area (µm^2^)	Sa (µm)	Sq (µm)	Sp (µm)	Sv (µm)	Sy (µm)	Average Height (µm)
Material (10 µm × 10 µm)	141.851	0.3358	0.4618	1.9228	−0.0648	1.9876	0.2449
Material (5 µm × 5 µm)	40.807	0.5047	0.5878	1.8672	−0.1181	1.9853	0.4144

**Table 3:** Textural parameters for SiO_2_/Fe(acac)_3_/NaF.

Sample	Surface Area, m^2^/g	Pore Size Distribution, DFT Method, nm	Total Pore Volume	Fhh Value
Material	276	5.564	3.940 e^−1^ cc/g for pores smaller than 130.1 nm	2.6644

**Table 4:** Kinetic parameters for the adsorption of As(III) onto SiO_2_/Fe(acac)_3_/NaF.

Pseudo-First Order Kinetic Model				
Temperature (K)	qe,exp (µg/g)	k_1_ (min^−1^)	qe,calc (µg/g)	R^2^
298	24.62	0.0564	10.05	0.9076
308	24.84	0.0624	11.39	0.8437
318	24.93	0.0645	13.07	0.7943
328	24.98	0.0768	19.00	0.8333
Pseudo-second order kinetic model				
Temperature (K)	qe,exp (µg g^−1^)	k_2_ (g µg^−1^∙min^−1^)	qe,calc (µg g^−1^)	R^2^
298	24.62	661.5	19.60	0.9930
308	24.84	975.8	21.27	0.9952
318	24.93	1601.7	26.11	0.9951
328	24.98	2727.2	26.25	0.9973
Intraparticle diffusion model				
Temperature (K)	k_diff_ (mg·g^−1^ min^−1/2^)	C		R^2^
298	1.32	6.71		0.8492
308	1.78	9.05		0.8373
318	1.84	9.58		0.8019
328	2.10	14.01		0.8345

**Table 5:** Thermodynamic parameters for adsorption of As(III) onto SiO_2_/Fe(acac)_3_/NaF.

ΔH° (kJ/mol)	ΔS° (J/mol∙K)	ΔG° (kJ/mol)				R^2^
68.56	254.6	298 K	308 K	318 K	328 K	0.9906
		−75.67	−78.21	−80.75	−83.29	

**Table 6:** Parameters of isotherm model for adsorption of As(III) onto SiO_2_/Fe(acac)_3_/NaF.

Langmuir Isotherm			
qm,exp (µg/g)	K_L_ (L/µg)	q_L_ (µg/g)	R^2^
574.9	0.067	650.28	0.9711
Freundlich isotherm			
K_F_ (µg/g)	1/n_F_		R^2^
58.61	0.31		0.8065
Sips isotherm			
K_S_	q_S_ (µg/g)	1/n_S_	R^2^
6.21	591.9	0.56	0.9916

**Table 7:** Comparison of adsorption performance with other material for As(III) adsorption.

Materials	q, µg/g	References
Allyl alcohol treated chicken feathers	0.115	[64]
TrisilanolCyclohexyl treated chicken feathers	0.110	[64]
Aluminum oxide NPs	0.500	[65]
Iron oxide-coated sand	0.029	[66]
Activated alumina	0.180	[67]
Modified chicken feathers	0.130	[64,68].
Rice polish	0.140	[69]
SiO_2_/Fe(acac)_3_/NaF	575.100	This paper

**Table 8:** Water composition after adsorption process.

Parameters	Content
Ca^2+^ (mg/L)	8.90 ± 0.89
Mg^2+^ (mg/L)	41.20 ± 4.12
Na^+^ (mg/L)	119.00 ± 11.9
K^+^ (mg/L)	0.85 ± 0.08
Fe^n+^ (mg/L)	0.20 ± 0.02
Mn^n+^ (mg/L)	0.03 ± 0.003
P_total_, (mg/L)	2.60 ± 0.26
${\mathrm{NH}}_{4}^{+}$, (mg/L)	2.40 ± 0.24
${\mathrm{NO}}_{3}^{-}$, (mg/L)	11.30 ± 1.13
${\mathrm{NO}}_{2}^{-}$, (mg/L)	0.34 ± 0.03
${\mathrm{SO}}_{4}^{2-}$, (mg/L)	4.20 ± 0.42
As(III) (μg/L)	9.30 ± 0.93
